# An explainable and efficient deep learning framework for EEG-based diagnosis of Alzheimer's disease and frontotemporal dementia

**DOI:** 10.3389/fmed.2025.1590201

**Published:** 2025-07-15

**Authors:** Waqar Khan, Muhammad Shahbaz Khan, Sultan Noman Qasem, Wad Ghaban, Faisal Saeed, Muhammad Hanif, Jawad Ahmad

**Affiliations:** ^1^Department of Cybersecurity, Pakistan Navy Engineering College, National University of Sciences and Technology, Karachi, Pakistan; ^2^School of Computing, Engineering and the Built Environment, Edinburgh Napier University, Edinburgh, United Kingdom; ^3^Computer Science Department, College of Computer and Information Sciences, Imam Mohammad Ibn Saud Islamic University (IMSIU), Riyadh, Saudi Arabia; ^4^King Salman Center for Disability Research, Riyadh, Saudi Arabia; ^5^Applied College, University of Tabuk, Tabuk, Saudi Arabia; ^6^College of Computing, Birmingham City University, Birmingham, United Kingdom; ^7^Department of Informatics, School of Business, Örebro Universitet, Örebro, Sweden; ^8^Cybersecurity Center, Prince Mohammad Bin Fahd University, Al-Khobar, Saudi Arabia

**Keywords:** explainable AI, XAI, Alzheimer's disease, temporal convolutional networks, long short-term memory, frontotemporal dementia, EEG, mental disorders

## Abstract

The early and accurate diagnosis of Alzheimer's Disease and Frontotemporal Dementia remains a critical challenge, particularly with traditional machine learning models which often fail to provide transparency in their predictions, reducing user confidence and treatment effectiveness. To address these limitations, this paper introduces an explainable and lightweight deep learning framework comprising temporal convolutional networks and long short-term memory networks that efficiently classifies Frontotemporal dementia (FTD), Alzheimer's Disease (AD), and healthy controls using electroencephalogram (EEG) data. Feature engineering has been conducted using modified Relative Band Power (RBP) analysis, leveraging six EEG frequency bands extracted through power spectrum density (PSD) calculations. The model achieves high classification accuracies of 99.7% for binary tasks and 80.34% for multi-class classification. Furthermore, to enhance the transparency and interpretability of the framework, SHAP (SHapley Additive exPlanations) has been utilized as an explainable artificial intelligence technique that provides insights into feature contributions.

## 1 Introduction

Frontotemporal dementia (FTD) (1) and Alzheimer's disease (2) (AD) are two most prevalent forms of dementia, primarily affecting individuals over 40 years of age. The global prevalence of dementia is expected to reach more than 130 million cases by 2050 (3). The rise in cases related to these diseases have significantly strained healthcare systems around the world, necessitating an urgent need for accurate and early diagnostic methods. The diagnosis of (FTD) and AD relies on the methodologies, such as neuropsychological evaluations (4), biomarkers analysis (5), established clinical criteria (6), and magnetic resonance imaging (MRI) (7). But the time requirements, need for expert interpretation, limit the practicality of advanced neuroimaging tools, and the high cost. Therefore, there is a critical need for early and accurate diagnosis, there is an indispensable need for improved detection methods. Timely diagnosis is critical, as early intervention can help slow disease progression and enhance patients' quality of life.

Electroencephalograms (EEG) offer features such as high temporal resolution, lower cost, and real-time monitoring, which make them valuable for dementia diagnosis. EEG signals in conjunction with machine learning, hold tremendous potential to be an effective non-invasive method to detect and monitor (FTD) and AD (8). However, extracting features from EEG is a crucial task, and although various methods have been proposed in research (9, 10), many of them have not achieved high accuracies with deep learning and machine learning models. Therefore, novel and tailored approaches are needed to extract high-quality data from EEG for improved analysis and diagnosis based on deep learning.

Deep learning (DL) models have shown significant potential in classifying EEG data, offering improved accuracy and efficiency in analysis. However, there is a need for lightweight models to optimize data processing and develop a high-performing model that is time-efficient, and computationally less loaded. In addition, most ML and DL models function as “black boxes,” providing outputs without transparency, which limits their acceptance, especially in sensitive fields like healthcare. Explainable Artificial Intelligence (XAI) offers a solution by revealing what the models learn during training and how decisions are made during prediction, making the results more understandable and interpretable. The core contributions of this research are given below.

- This research introduces an EEG-based feature extraction approach using modified Relative Band Power (RBP) analysis for feature engineering and proposes a lightweight hybrid deep learning classifier for accurate and robust classification of frontotemporal dementia, Alzheimer's disease, and health.
- SHAP (SHapley Additive Explanations), an explainable artificial intelligence technique has been integrated into the model to provide deeper insights into feature contributions, increasing interpretability, transparency, and prediction reliability for mental disorder diagnosis.

This is how the rest of the article is organized. Related work is covered in Section 2, and methodology is covered in Section 3. Our research findings are shown in Section 4, and explainable artificial intelligence is covered in Section 5. Section 6 concludes with a summary of our findings and recommendations for future research.

## 2 Related work

Recent studies have focused on enhancing the Alzheimer's disease detection with advanced machine learning methods. To solve supervised AD detection using EEG data analysis, machine, and deep learning-based systems have gained popularity (11–13). The study (14) used a public EEG signal dataset that included recordings from 12 Alzheimer's disease patients and 11 healthy controls. A directed graph approach was applied for local texture feature extraction, resulting in 448 low-level features per EEG signal. This was further enhanced by combining it with a tunable q-factor wavelet transform, resulting in a total of 8,512 features per signal input. The accuracy of the model was 92.01% with leave-one-subject-out (LOSO) cross-validation and 100% with 10fold cross-validation.

Moreover, six supervised machine-learning approaches were used in this work (15) to categorize processed EEG data from patients with FTD and AD. Different techniques for processing and analyzing EEG signals were applied to identify relevant features.The accuracy of the decision tree machine learning model was 78.5%, while the random forest model attained an accuracy of 86.3% in diagnosing FTD. This study (16) proposes a convolutional neural network-based model called STEADYNet, which achieves high performance with 98.24% accuracy in dementia detection using multichannel spatiotemporal EEG signals.

Another study (17) proposes a CNN-based model utilizing the Forward-Backward Fourier Transform (FBFT) to enhance EEG signal visualization for brain disorder classification. The model achieves 85.1% for murmur, 99.82% accuracy for epilepsy, 100% for mental stress, and 95.91% for Alzheimer's disease (AD). Additionally, the eye-naked classification approach attains 78.6%, 71.9%, 82.7%, and 91.0% accuracy for epilepsy, AD, murmur, and mental stress, respectively.

In addition, a study (18) offers a “dual-input convolution encoder network” as a unique method for classifying AD. Denoising and the extraction of band power and coherence characteristics from the EEG data were important feature engineering approaches. With an accuracy of 83.28% in differentiating AD patients from healthy controls, the presented model combines convolutional layers with transformer architecture, and feed-forward module and proves its efficacy in collecting intricate EEG features.

## 3 Methodology

### 3.1 Data collection

**Figure 1:**
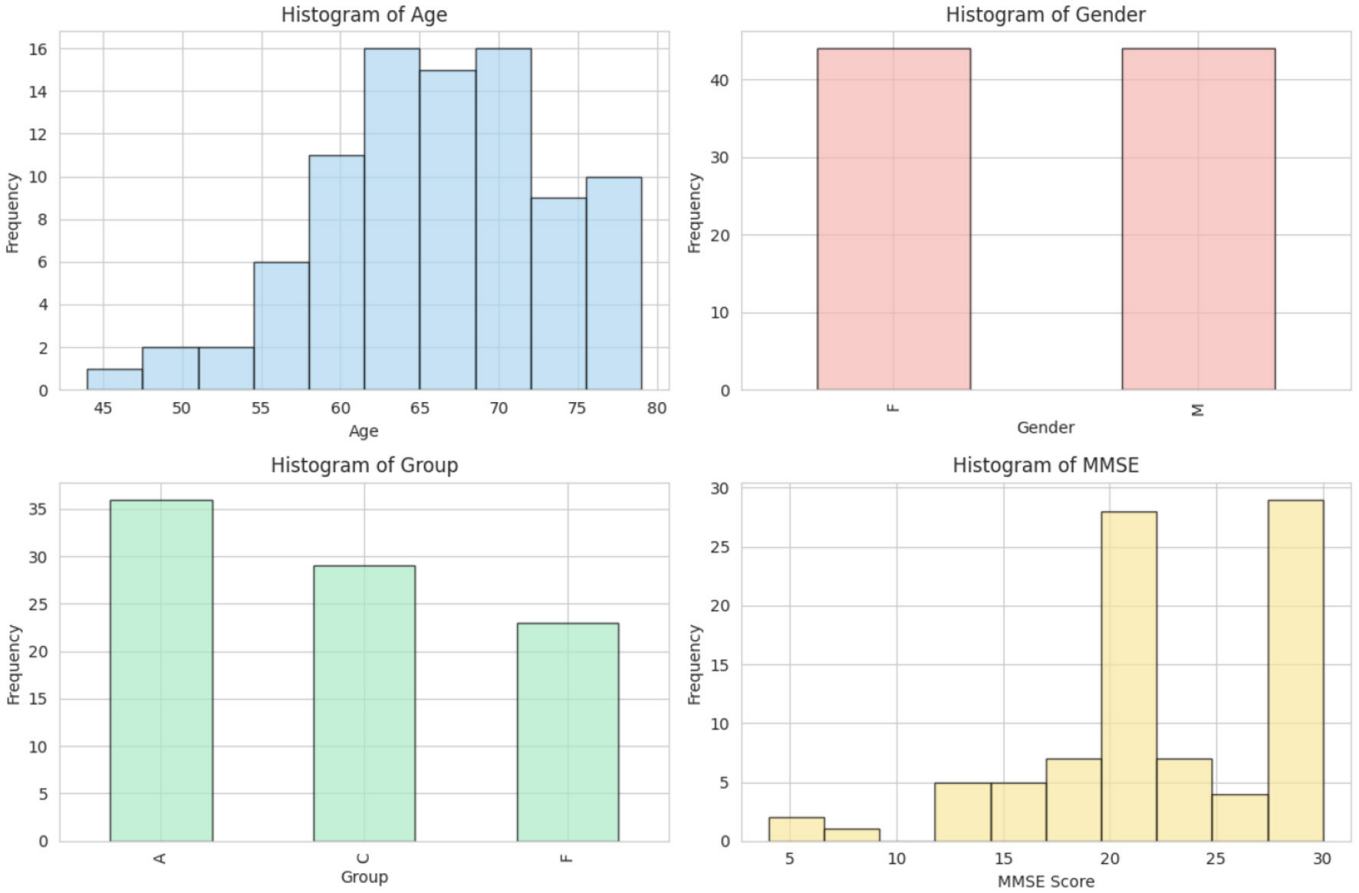
Statistical overview of the dataset.

The dataset (8) consists of EEG recordings from 88 subjects (36 Alzheimer's disease, 29 healthy and 23 frontotemporal dementia) obtained at the 2nd Neurology Department of AHEPA General University Hospital, and data statistics as shown in Figure 1. EEG signals were captured using 19 electrodes while participants remained seated with their eyes closed. The data was initially filtered at 0.5–60 Hz and sampled at 500 Hz.

### 3.2 Data preprocessing

**Figure 2:**
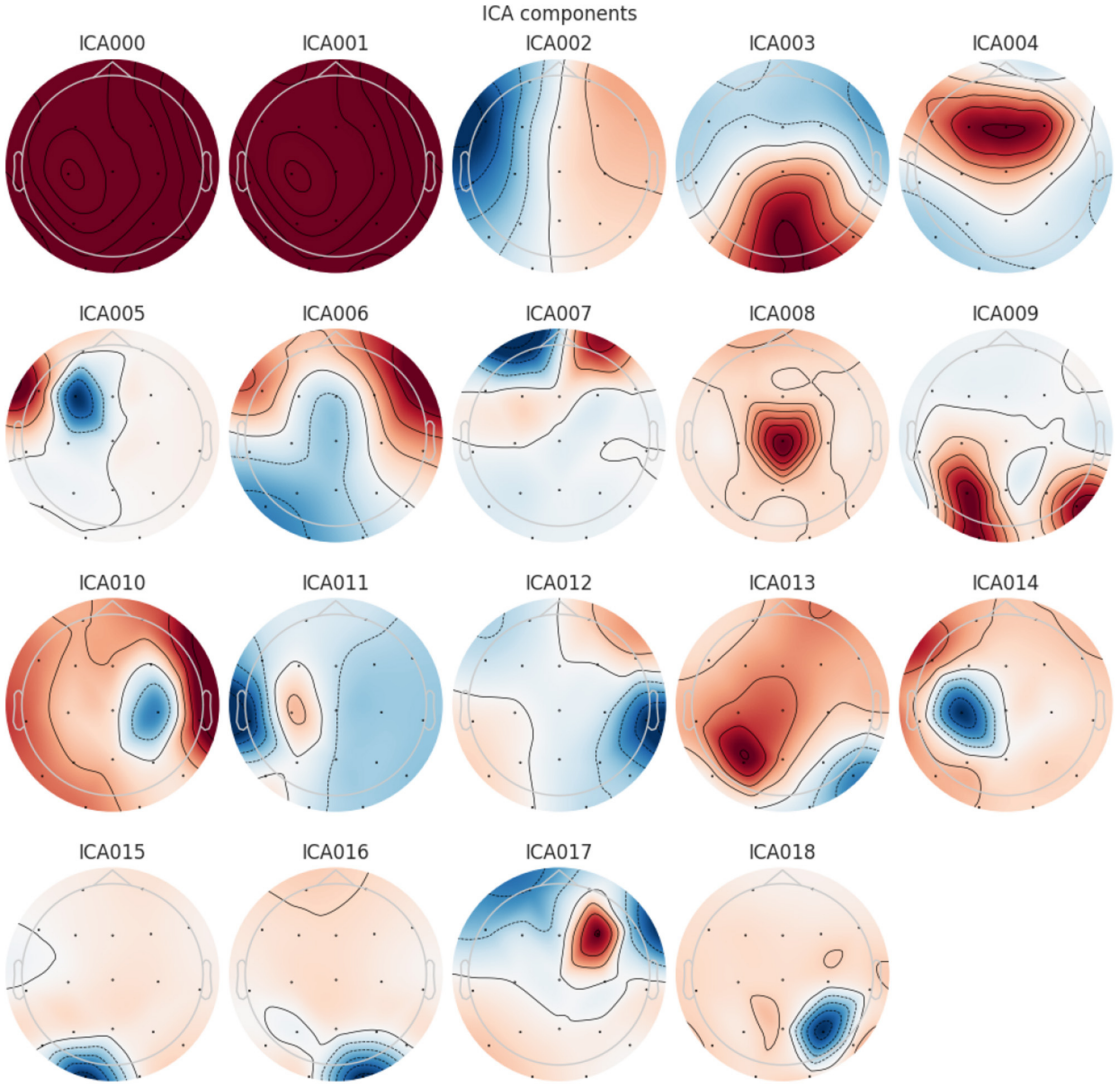
Independent Component Analysis (ICA) components extracted from EEG signal.

To enhance the quality of the electroencephalogram (EEG) signals and remove unwanted artifacts, a systematic pre-processing technique has been applied. Initially, a Butterworth bandpass filter with a frequency range of 0.5 Hz to 45 Hz was used to retain relevant neural activity while eliminating low-frequency drifts and high-frequency noise. Next, Artifact Subspace Reconstruction (ASR) was implemented to identify and correct signal distortions. ASR detects artifacts by measuring the standard deviation of signal segments within a 0.5-s window. Segments exceeding a deviation threshold of 17 were reconstructed to suppress transient artifacts while preserving the integrity of neural activity. After the artifact correction, Independent Component Analysis (ICA) was performed using the RunICA algorithm. This process decomposed the 19-channel EEG signals into independent components, as illustrated in Figure 2. The independent components were then analyzed using EEGLAB's ICLabel tool, which automatically classifies components based on their source characteristics. Components identified as “eye artifacts” or “jaw artifacts” were removed to ensure that only neural activity remained in the processed signals. Although EEG signals were recorded in a closed-eye resting state, some residual eye movement artifacts were still present. The implemented pre-processing steps effectively mitigated these unwanted influences, ensuring cleaner EEG signals for subsequent analysis.

### 3.3 Feature engineering

In EEG classification tasks, relative band power (RBP) (15) is often extracted, especially when analyzing brain activity related to various neurological and cognitive states. The RBP is calculated for several frequency bands that correspond to various facets of brain activity. Six interesting frequency bands were taken into consideration in this study:

- **Delta:** 0.5 ≤ *f* < 4Hz
- **Theta:** 4 ≤ *f* < 8Hz
- **Alpha:** 8 ≤ *f* < 16Hz
- **Zaeta:** 16 ≤ *f* < 24Hz
- **Beta:** 24 ≤ *f* < 30Hz
- **Gamma:** 30 ≤ *f* ≤ 45Hz.

The Welch technique is used to compute the Power Spectral Density by a given equation


(1) $$\begin{array}{l} \text{PSD}\left(f\right)=\underset{N\to \infty }{\lim}\frac{1}{N}\overset{N-1}{\underset{n=0}{\sum }}|X\left(f_{n}\right){|}^{2} \end{array}$$

where *X*(*f*_*n*_) is the Fourier transform of the signal *x*(*t*) evaluated at frequency bins *f*_*n*_, and *N* is the total number of segments over which the Fourier transform is averaged. The overall power in the frequency range of 0.5–45 Hz is calculated by summing the PSD values.


(2) $$\begin{array}{l} \text{Total PSD}=\overset{\text{max}}{\underset{f=\text{min}}{\sum }}\text{PSD}\left(f\right) \end{array}$$

The RBP for each frequency band *b* is determined by dividing the power within the band by the overall power.


(3) $$\begin{array}{l} {\text{RBP}}_{b}=\frac{\sum_{f=\text{min}}^{f_{\text{max}}}\text{PSD}\left(f\right)}{\sum_{f=0.5}^{45}\text{PSD}\left(f\right)} \end{array}$$

The power in the frequency band [*f*_min_, *f*_max_] is represented by the numerator, while the total power in the range of 0.5 Hz to 45 Hz is the denominator.

These bands provide greater in-depth observations and cover a wider range of brain activity. In order to compute the RBP, EEG signals are segmented into epochs, each 6 s in length and sharing a 50% overlap. By splitting the signal into overlapping segments, calculating the squared magnitude of the discrete Fourier transform for each segment, and then averaging the results, the Welch technique is used to estimate the Power spectral Density for each epoch. After that, the relative power inside each frequency band is determined by dividing the PSD for that band by the PSD for the whole frequency range of interest 0.5–45 Hz. A normalized measure of brain activity is provided by this ratio, which shows the contribution of each frequency band to the signal's overall strength. For each epoch, the RBP is computed across all channels:


(4) $$\begin{array}{l} \text{Epoch RBP}=\frac{1}{N_{\text{channels}}}\overset{N_{\text{channels}}}{\underset{i=1}{\sum }}{\text{RBP}}_{b}\left(i\right) \end{array}$$

where RBP_*b*_(*i*) is the RBP for the *i*-th channel and *N*_channels_ is the number of EEG channels.

The RBP values for every epoch make up the final feature matrix. The columns match the six frequency bands (Beta, Delta, Alpha, Theta, Zaeta, and Gamma), whereas each row denotes an epoch:


$$\text{Feature Matrix}=\left[\begin{array}{ccccccc} \text{Delta} & \text{Theta} & \text{Alpha} & \text{Zaeta} & \text{Beta} & \text{Gamma} & \text{Label} \end{array}\right]$$

Once the RBP features have been extracted, they are used as inputs for classification tasks. Each epoch is labeled according to whether the person has frontotemporal dementia, Alzheimer's disease, or cognitive normal.

### 3.4 Label encoding and data normalization and splitting

The data was saved in a comma-separated file, and then categorical variables were converted to numerical data using one-hot encoding. Then, the data was normalized using the min-max normalization formula given by:


(5) $$\begin{array}{l} \chi^{*}=\frac{\chi -\mu_{\min}}{\mu_{\max}-\mu_{\min}} \end{array}$$

The normalized value is represented by χ^*^, the original value is represented by χ, and the dataset's minimum and maximum values are indicated by μ_min_ and μ_max_, respectively. Training, validation, and test data sets were split into 80%, 10%, and 10% of the total data set.

### 3.5 The proposed deep learning model

**Figure 3:**
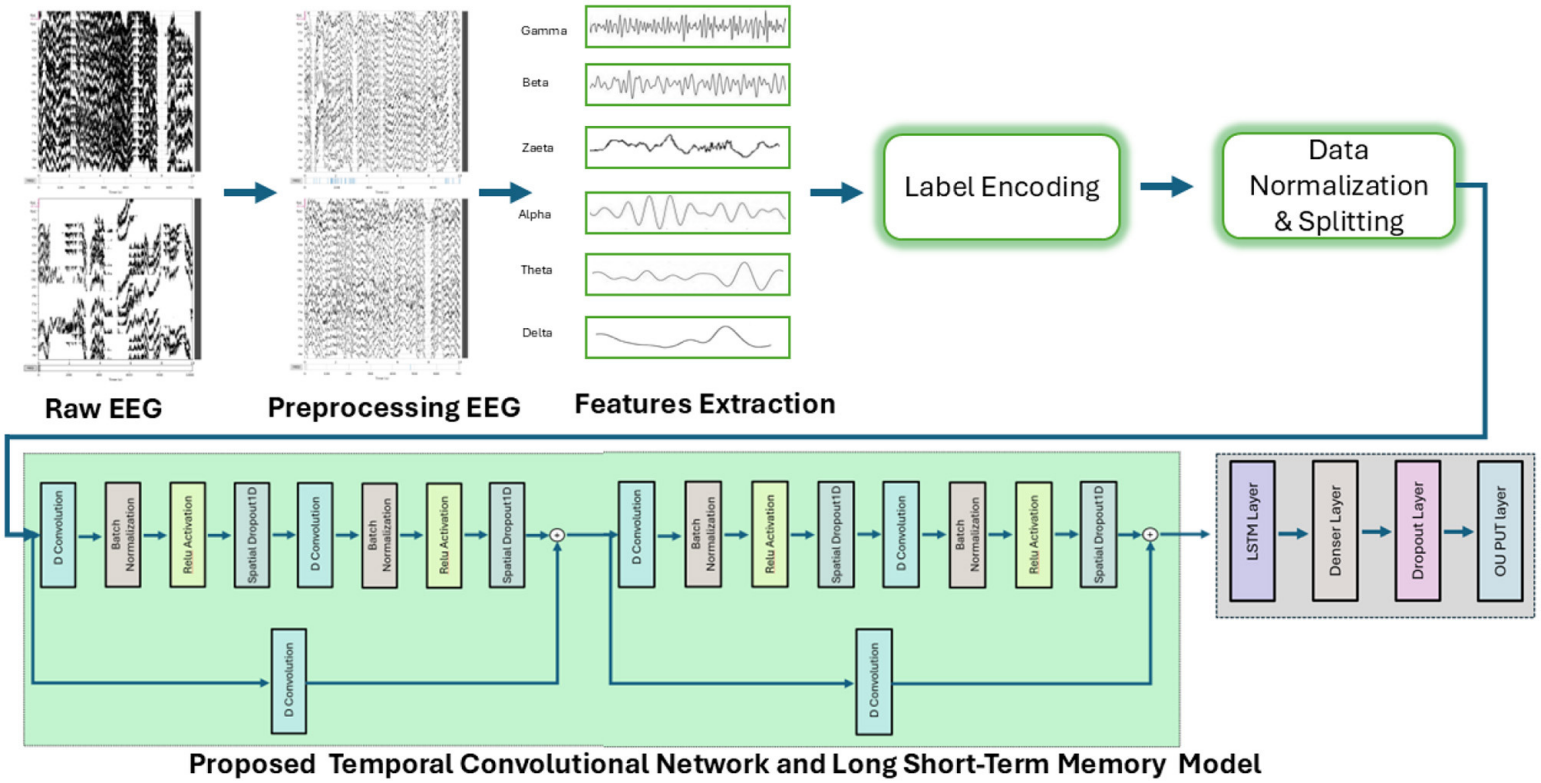
The proposed methodology with the proposed deep learning model.

The proposed hybrid model as given in Figure 3. Its consists of two deep learning components LSTM and TCN. The TCN uses dilated causal convolutions to obtain high-level features from the input sequence, and the LSTM captures the sequential dependencies. The Temporal Convolutional Network enhances traditional CNNs with dilated causal convolutions, allowing them to model long-term temporal patterns without violating sequence order.


(6) $$\begin{array}{l} H^{\left(l\right)}=\sigma \left(W^{\left(l\right)}*X+b^{\left(l\right)}\right) \end{array}$$

where *H*^(*l*)^ represents the output of the *l*-th convolutional layer, the learnable convolutional filters are represented by *W*^(*l*)^, the convolution operation is represented by *, the bias is represented by *b*^(*l*)^, and the ReLU activation function is represented by σ(·).

Long-range interdependence in EEG data can be captured with the use of dilated convolutions:


(7) $$\begin{array}{l} H_{t}^{\left(l\right)}=\overset{k-1}{\underset{i=0}{\sum }}W_{i}^{\left(l\right)}\cdot X_{t-d\cdot i}+b^{\left(l\right)} \end{array}$$

where *k* is the kernel size and *d* is the dilation rate. To optimize both stability and the flow of gradients, residual connections are adopted.


(8) $$\begin{array}{l} H_{\text{res}}^{\left(l\right)}=H^{\left(l\right)}+X \end{array}$$

This structure enables efficient learning without vanishing gradients.

LSTMs are a unique class of recurrent neural networks that use gate mechanisms and memory cells to manage long-term dependencies. The LSTM uses three primary gates—forget, input, and output gates to process the features that were extracted from TCN.


(9) $$\begin{array}{l} f_{t}=\sigma \left(W_{f}H_{t}^{\left(l\right)}+U_{f}h_{t-1}+b_{f}\right) \end{array}$$

(10) $$\begin{array}{l} i_{t}=\sigma \left(W_{i}H_{t}^{\left(l\right)}+U_{i}h_{t-1}+b_{i}\right) \end{array}$$

(11) $$\begin{array}{l} c_{t}^{\prime }=\tanh\left(W_{c}H_{t}^{\left(l\right)}+U_{c}h_{t-1}+b_{c}\right) \end{array}$$

(12) $$\begin{array}{l} c_{t}=f_{t}⊙c_{t-1}+i_{t}⊙c_{t}^{\prime } \end{array}$$

(13) $$\begin{array}{l} o_{t}=\sigma \left(W_{o}H_{t}^{\left(l\right)}+U_{o}h_{t-1}+b_{o}\right) \end{array}$$

(14) $$\begin{array}{l} h_{t}=o_{t}⊙\tanh\left(c_{t}\right) \end{array}$$

where *W*_*f*_ the input's weight matrix at time step *t*. The input to the LSTM at layer *l* and time *t* is represented by the item $H_{t}^{\left(l\right)}$. *U*_*f*_ Weight matrix for the preceding time step's hidden state. *h*_*t*−1_ The previously hidden state. Adding the bias term *b*_*f*_ and the sigmoid activation function σ.

The model begin with an input layer shaped (6,1), followed by a 1D convolutional layer with 32 filters as shown in Table 1. The first layer connects to a batch normalization layer having 128 number of parameters and an activation function, then goes through a spatial dropout layer having value 0.2. The next convolutional layer also uses 32 filters, followed by batch normalization and another activation layer. A residual connection is created by adding the output of a separate convolution layer with the same shape, allowing the model to retain important features. Furthermore, a similar set of layers is added next, helping the model process the input in the same way as before. The model uses an LSTM layer with 64 units to capture temporal features. Following this, the model includes a dense layer with 128 units, which is succeeded by two additional dense layers containing 192 and 256, units respectively; each of these layers is paired with a dropout mechanism to help mitigate overfitting, culminating in a final dense layer with 3 output units that delivers the classification outcome.

**Table 1:** Model architecture summary.

**Layer (type)**	**Output shape**	**Parameters**	**Connected to**
Input layer	(None, 6, 1)	0	-
Conv 1D	(None, 6, 32)	256	Input layer [0][0]
Batch normalization	(None, 6, 32)	128	Conv1D [0][0]
Activation	(None, 6, 32)	0	Batch normalization [0][0]
Spatial dropout 1D	(None, 6, 32)	0	Activation [0][0]
Conv1D	(None, 6, 32)	7,200	Spatial dropout 1D [0][0]
Batch normalization	(None, 6, 32)	128	Conv1D [1][0]
Activation	(None, 6, 32)	0	Batch normalization [1][0]
Conv 1D	(None, 6, 32)	64	Input layer [0][0]
Spatial dropout 1D	(None, 6, 32)	0	Activation [1][0]
Add	(None, 6, 32)	0	Conv1D[2][0], Spatial dropout 1D [1][0]
Conv 1D	(None, 6, 32)	7,200	Add[0][0]
Batch normalization	(None, 6, 32)	128	Conv1D [3][0]
Activation	(None, 6, 32)	0	Batch normalization [2][0]
Spatial dropout 1D	(None, 6, 32)	0	Activation [2][0]
Conv 1D	(None, 6, 32)	7,200	Spatial dropout 1D [2][0]
Batch normalization	(None, 6, 32)	128	Conv 1D [4][0]
Activation	(None, 6, 32)	0	Batch normalization [3][0]
Conv 1D	(None, 6, 32)	1,056	Add[0][0]
Spatial dropout 1D	(None, 6, 32)	0	Activation [3][0]
Add	(None, 6, 32)	0	Conv1D[5][0], Spatial dropout 1D [3][0]
LSTM	(None, 64)	24,832	Add [1][0]
Dense	(None, 128)	8,320	LSTM [0][0]
Dropout	(None, 128)	0	Dense [0][0]
Dense	(None, 192)	24,768	Dropout [0][0]
Dropout	(None, 192)	0	Dense [1][0]
Dense	(None, 256)	49,408	Dropout [1][0]
Dropout	(None, 256)	0	Dense [2][0]
Dense	(None, 3)	771	Dropout [2][0]

### 3.6 Hyperparameter tuning

Random search-based hyperparameter tuning was used to find the optimal number of layers in the proposed model. The best hyperparameter values for the CNN component are: two TCN blocks, 32 filters, a kernel size of 7, a dropout rate of 0.3, and a dilation rate of 1. During optimization, the best LSTM structure was found to be a single layer of 64 units. Dense layers follow with 128, 192, and 256 units and a 0.2 dropout rate and early stopping mitigates overfitting. The number of training epochs depended on the specific classification task. A batch size of 32 was used, and the Adam optimizer was selected with a learning rate of 0.0001. The model has 131,587 parameters. it uses 514.01 KB of memory, making it suitable for deployment on edge medical devices for real-time mental disorder detection. Out of these, 131,331 are trainable and 256 are non-trainable as shown in the Table 2. The model was trained using 8 GB RAM, and each epoch took 6 s.

**Table 2:** Model parameter summary.

**Parameter type**	**Count**	**Size**
Total parameters	131,587	514.01 KB
Trainable parameters	131,331	513.01 KB
Non-trainable parameters	256	1 KB

### 3.7 Classification

The proposed hybrid Temporal Convolutional Network model with Long Short-Term Memory was utilized to perform four types of classification tasks for Alzheimer's Disease, Frontotemporal Disease, and healthy classes. The classification tasks are as follows:

- **Classification for Alzheimer's, frontotemporal, and healthy classes:**the objective of this work was to categorize three different classes: healthy controls, frontotemporal disease, and Alzheimer's disease. The model was trained to distinguish between the three groups.
- **Classification for Alzheimer + frontotemporal disease and healthy classes:** in this classification the model was trained to classify a combined class of Alzheimer's Disease and Frontotemporal Disease from healthy individuals.
- **Classification for Alzheimer's disease and healthy classes:** the objective of this task is to train the model to classification between the Healthy class and Alzheimer's disease.
- **Classification for frontotemporal disease and healthy classes:** this classification task required the model to separate individuals with Frontotemporal Disease from healthy controls.

## 4 Results

### 4.1 Performance parameters

To access the performance of the proposed model, the key performance parameters, i.e., precision, F1 score, accuracy, recall, sensitivity, etc. have been extensively evaluated. Among other, accuracy is the most important performance parameter for assessing a classification model's efficacy. It measures the proportion of accurately predicted instances to all instances in the dataset, including true positives and negatives. Mathematically, accuracy can be written as:


(15) $$\begin{array}{c} \text{Accuracy}=\frac{\text{Number of Correct Predictions}}{\text{Total Number of Predictions}}\times 100 \end{array}$$

(16) $$\begin{array}{c} \text{Accuracy}=\frac{\text{TP}+\text{TN}}{\text{TP}+\text{TN}+\text{FP}+\text{FN}}\times 100\% \end{array}$$

Similarly, precision measures the quality of the model's prediction. It measures the percentage of properly identified positive cases in comparison to the total number of cases that were anticipated to be positive (sum of true positives and false positives). Precision can be shown mathematically as:


(17) $$\begin{array}{c} \text{Precision}=\frac{\text{Number of Correctly Predicted Positive Cases}}{\text{Total Predicted Positive Cases}}\times 100 \end{array}$$

(18) $$\begin{array}{c} \text{Precision}=\frac{\text{TP}}{\text{TP}+\text{FP}}\times 100\% \end{array}$$

Recall, also known as the true positive rate, is a crucial performance indicator that assesses how well a classification model detects positive. Recall can be mathematically represented as:


(19) $$\begin{array}{c} \text{Recall}=\frac{\text{Number of Correctly Identified Positive Cases}}{\text{Total Actual Positive Cases}}\times 100 \end{array}$$

(20) $$\begin{array}{c} \text{Recall}=\frac{\text{TP}}{\text{TP}+\text{FN}}\times 100 \end{array}$$

The F1 score offers a balance between accuracy and recall by taking the harmonic mean of the accuracy and recall metrics. It is particularly convenient when dealing with imbalanced datasets. Mathematically, the F1 score is expressed as:


(21) $$\begin{array}{c} \text{F1-score}=\frac{2\times \text{Precision}\times \text{Recall}}{\text{Precision}+\text{Recall}}\times 100 \end{array}$$

(22) $$\begin{array}{c} \text{F1-score}=\frac{2\times \frac{\text{TP}}{\text{TP}+\text{FP}}\times \frac{\text{TP}}{\text{TP}+\text{FN}}}{\frac{\text{TP}}{\text{TP}+\text{FP}}+\frac{\text{TP}}{\text{TP}+\text{FN}}}\times 100 \end{array}$$

Specificity measures the proportion of actual negatives that the model correctly identifies. It evaluates the model's ability to correctly identify true negatives.


(23) $$\begin{array}{c} \text{Specificity}=\frac{\text{Number of Correctly Identified Negative Cases}}{\text{Total Actual Negative Cases}}\times 100 \end{array}$$

(24) $$\begin{array}{c} \text{Specificity}=\frac{\text{TN}}{\text{TN}+\text{FP}}\times 100 \end{array}$$

In above equations, TP represents True Positives or correctly identified positive cases, TN represents True Negatives or correctly identified negative cases, FP represents False Positives or incorrectly classified as positive, and FN represents False Negatives or incorrectly classified as negative.

### 4.2 Performance evaluation

Table 3 presents the classification metrics for the three classes: Alzheimer, Frontotemporal, and Healthy.The model achieves 70% precision and 90% recall for Alzheimer's, with an F1-score of 0.79. It performs perfectly for frontotemporal Disease with 100% F1-score, precision, and recall, while the Healthy class shows weaker performance with 68% precision, 35% recall, and an F1-score of 0.47.

**Table 3:** Classification metrics for Alzheimer, frontotemporal, and healthy classes.

**Class**	**Precision**	**Recall**	**F1-score**	**Sensitivity**	**Specificity**	**Support**
Alzheimer	0.70	0.90	0.79	0.90	0.74	1,876
Frontotemporal	1.00	1.00	1.00	1.00	1.00	1,597
Healthy	0.68	0.35	0.47	0.35	0.95	1,106

Table 4 focuses on a binary classification task where Alzheimer + Frontotemporal Disease are treated as a combined class, and Healthy is the other class.he model achieves nearly perfect results for Alzheimer + Frontotemporal Disease with 99.77% precision and 99.93% recall. The Healthy class also performs well with 99.87% precision and 99.56% recall, both classes showing 1.00 sensitivity and specificity.

**Table 4:** Classification metrics for Alzheimer + frontotemporal disease and healthy classes.

**Metric**	**Alzheimer + frontotemporal disease**	**Healthy**
Precision	0.9977	0.9987
Recall	0.9993	0.9956
F1 score	0.9985	0.9972
Support	2,983	1,596
Sensitivity	1.00	
Specificity	1.00	

In the binary classification task, the Table 5, the goal is to classify Alzheimer's disease and healthy individuals.The model excels with 99.63% precision and 99.89% recall for Alzheimer's, and 99.87% precision and 99.56% recall for Healthy, both showing 1.00 sensitivity and specificity.

**Table 5:** Classification metrics for Alzheimer's disease and healthy classes.

**Metric**	**Alzheimer's disease**	**Healthy**
Precision	0.9963	0.9987
F1 Score	0.9976	0.9972
Recall	0.9989	0.9956
Support	1876	1596
Sensitivity	1.00	
Specificity	1.00	

The Table 6 shows the results of the binary classification between frontotemporal disease and healthy individuals. The model shows 99.94% precision and 99.56% recall, with a very high F1 score of 0.9975. The Healthy class also has a high F1-score of 0.9964, with 99.37% precision and 99.91% recall. Both classes show 1.00 sensitivity and specificity. Similarly, Table 7 reports high classification accuracies for binary dementia tasks (≥0.997) and a lower accuracy (0.8034) for the three-class classification among Alzheimer's, frontotemporal disease, and healthy subjects.

**Table 6:** Classification metrics for frontotemporal disease and healthy classes.

**Metric**	**Frontotemporal disease**	**Healthy**
F1 score	0.9975	0.9964
Recall	0.9956	0.9991
Precision	0.9994	0.9937
Support	1597	1596
Sensitivity	1.00	
Specificity	1.00	

**Table 7:** Classification accuracy for different dementia classification tasks.

**Classification task**	**Accuracy**
Frontotemporal disease vs. healthy	0.9970
Alzheimer's disease vs. healthy	0.9974
Alzheimer + frontotemporal disease vs. healthy	0.9980
Alzheimer vs. frontotemporal vs. healthy	0.8034

**Figure 4:**
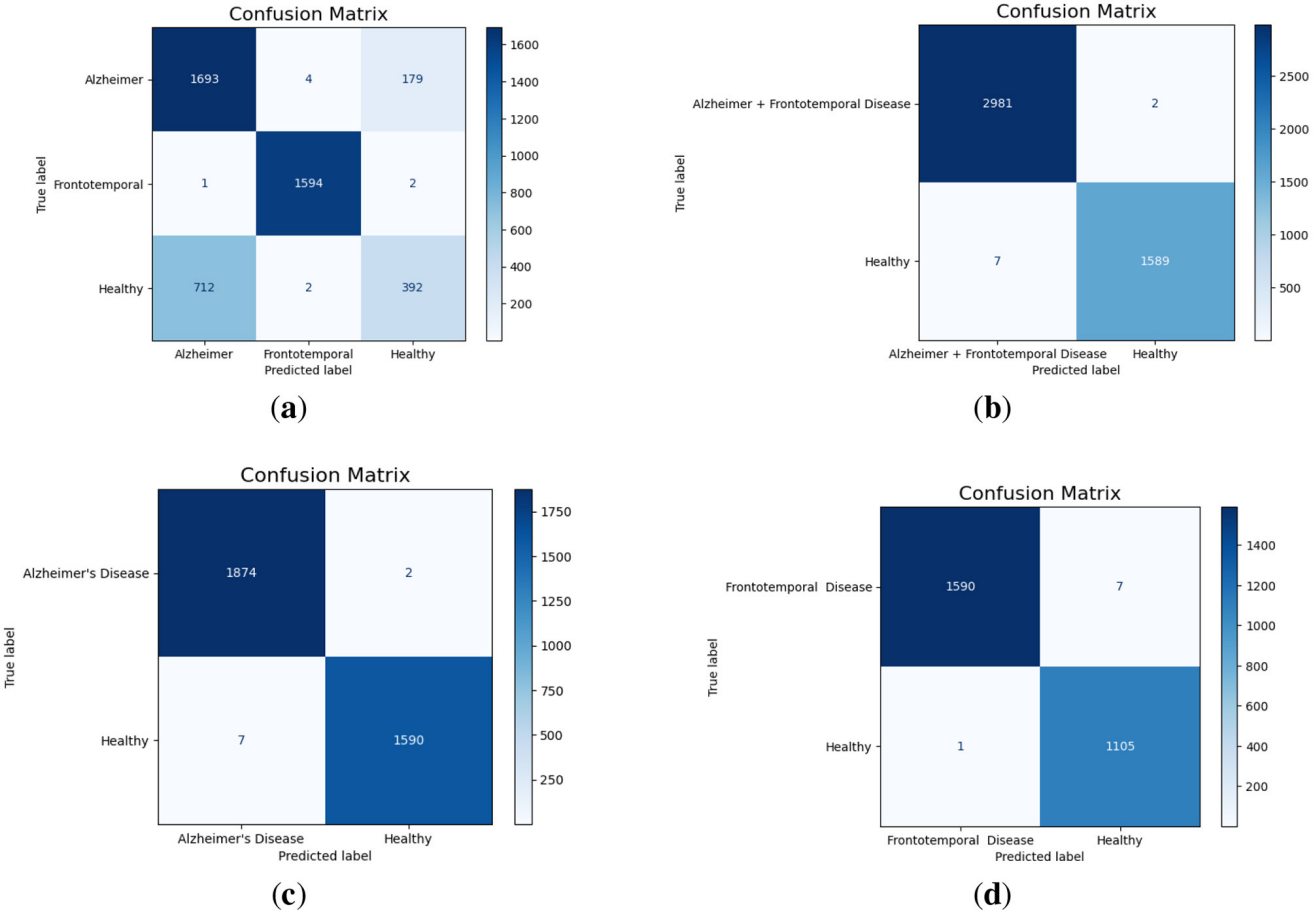
Confusion matrices for different classification scenarios. **(a)** Alzheimer vs. Frontotemporal vs. Healthy. **(b)** Alzheimer + Frontotemporal vs. Healthy. **(c)** Alzheimer vs. Healthy. **(d)** Frontotemporal vs. Healthy.

The multi-class confusion matrix given in Figure 4a that the model effectively classifies Alzheimer's and frontotemporal Disease. Out of 1,876 Alzheimer cases, 1,693 are correctly identified, with minor misclassifications into healthy and frontotemporal. Similarly, frontotemporal disease achieves a near-perfect classification with only three misclassifications. However, the model struggles significantly with Healthy cases, misclassifying 712 instances as Alzheimer's, highlighting room for improvement in distinguishing healthy from disease classes. When combining Alzheimer's and Frontotemporal as a single class against Healthy, the model demonstrates almost perfect classification as shown in Figure 4b. Only 2 out of 2,983 Alzheimer + frontotemporal instances are misclassified as healthy. For the Healthy class, only 7 out of 1,596 instances are misclassified, indicating strong model performance in binary classification with very few false positives or false negatives. For Alzheimer's Disease vs. Healthy classification, as displayed in Figure 4c, the model achieves excellent performance. Out of 1,876 Alzheimer cases, only 2 are misclassified as Healthy. Similarly, for 1,590 Healthy cases, only 7 are misclassified as Alzheimer. Furthermore, the model performs well in the binary classification of frontotemporal disease against healthy as evident from Figure 4d. Just one healthy case out of 1,106 is incorrectly categorized as frontotemporal, whereas only 7 out of 1,597 frontotemporal patients are incorrectly classified as healthy.

**Figure 5:**
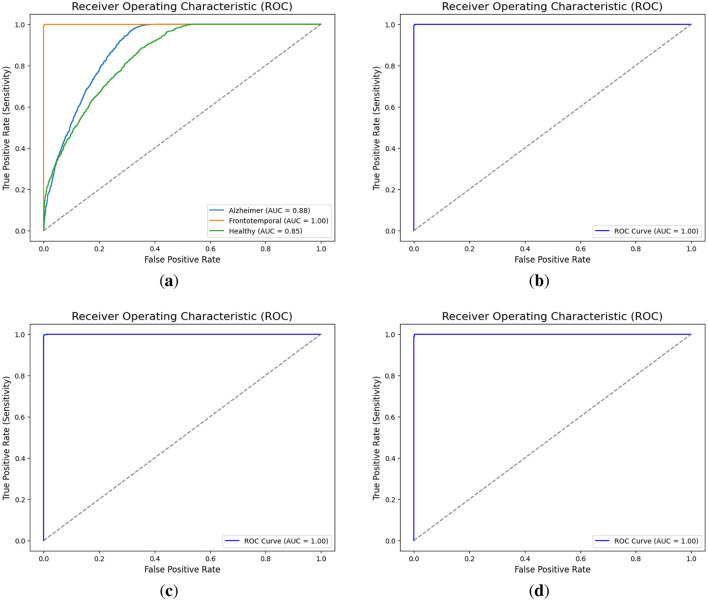
AUC curves for different classification scenarios. **(a)** Alzheimer vs. Frontotemporal vs. Healthy. **(b)** Alzheimer + Frontotemporal vs. Healthy. **(c)** Alzheimer vs. Healthy. **(d)** Frontotemporal vs. Healthy.

The multi-class ROC (Receiver Operating Characteristic) curve, as given in Figure 5a, displays the AUC (Area Under the Curve) for each class. Alzheimer's disease has an AUC of 0.88, which indicates good but not perfect discrimination; healthy cases have the lowest AUC of 0.85, which indicates some difficulty in differentiating them from the disease classes; and frontotemporal disease achieves a perfect AUC of 1.00, which indicates ideal classification with no false positives or negatives. The binary classification combining Alzheimer's and frontotemporal as one class vs. healthy achieves an exceptional AUC of 1.00. Additionally, the model attains an AUC of 1.00 for Alzheimer's Disease vs. Healthy cases, indicating perfect discrimination. Similarly, the classification of ‘Alzheimer + Frontotemporal' vs. Healthy, Alzheimer vs. Healthy, and Frontotemporal Disease vs. Healthy achieve a flawless AUC of 1.00 as displayed in Figures 5b–d, respectively.

### 4.3 Model performance evaluation with SMOTE balancing

It was noted in all the classification task that the dataset was imbalanced. To address this issue Smote data balancing technique were used. SMOTE balances datasets by generating new samples along the lines connecting a minority instance and its nearest within-class neighbors. Table 8 shows the classification metrics for Alzheimer, Frontotemporal, and Healthy classes after applying data balancing techniques. It can see a significant improvement in F1-score, precision, recall and specificity for all classes. Frontotemporal class got perfect scores in all metrics (1.00). The Alzheimer's class got good scores with precision 0.63, recall 0.71 and F1-score 0.67. Healthy class got precision 0.67, recall 0.58 and F1-score 0.62. Overall accuracy of the model decreased to 77.45% after balancing compared to 80.34% accuracy with the original imbalanced dataset.

**Table 8:** Classification metrics for Alzheimer, frontotemporal, and healthy classes with data balancing.

**Class**	**Precision**	**Recall**	**F1-score**	**Sensitivity**	**Specificity**	**Support**
Alzheimer	0.63	0.71	0.67	0.71	0.79	1,876
Frontotemporal	1.00	1.00	1.00	1.00	1.00	1,876
Healthy	0.67	0.58	0.62	0.58	0.86	1,876

The classification metrics for the Alzheimer's Disease and Healthy classes are shown in Table 9 after data balancing. The model ability to distinguish between the two classes is demonstrated by the precision, recall, and F1 scores of both classes, all of which are above 99.7%. Even though the balanced model's accuracy is 99.71%, it is only slightly lower than the unbalanced model's 99.74% accuracy.

**Table 9:** Classification metrics for Alzheimer's disease and healthy classes with data balancing.

**Metric**	**Alzheimer's disease**	**Healthy**
Precision	99.73	99.70
F1 score	99.71	99.73
Recall	99.70	99.71
Support	1876	1876

### 4.4 Evaluation of model accuracy using K-fold cross-validation

In this paper, a 5-fold cross-validation methodology was employed to validate the proposed model. The dataset was split into five subsets. For the multiclass classification task, the training accuracy ranged from a minimum of 79.43% to a maximum of 81.27% across different K values. The test accuracy remained consistently close to 80% for all folds, as shown in the Table 10. Table 11 shows the 5-fold cross-validation findings for differentiating between Alzheimer's and healthy patients. The test accuracy remains the same as in the training accuracy. These findings demonstrate the model's robust and reliable capacity to differentiate between the Alzheimer's and healthy classes.

**Table 10:** K-fold validation accuracy for Alzheimer, frontotemporal, and healthy classes.

**K-value**	**Training accuracy (%)**	**Test accuracy (%)**
1	79.89	80.15
2	80.00	80.00
3	79.58	80.06
4	79.43	80.02
5	81.27	80.13

**Table 11:** K-fold validation accuracy for Alzheimer and healthy classes

**K-value**	**Training accuracy (%)**	**Test accuracy (%)**
1	99.82	99.86
2	99.80	99.82
3	99.73	99.92
4	99.61	99.86
5	99.78	99.82

### 4.5 Comparative analysis of feature extraction methods

In this evaluation, we compared the standard RBP with our modified RBP. The same methodology was used, but the frequency ranges were adjusted according to the standard: Delta (0.5–4), Theta (4–8), Alpha (8–13), Beta (13–25), and Gamma (25–45). The results achieved are shown in the Table 12. The standard RBP method achieved an accuracy of 63.03% in the multiclass classification task, whereas the modified RBP reached 80.34%. The precision for all classes remained almost the same; however, the recall and F1-score varied across the three classes. The Alzheimer class showed higher F1-score and recall values, whereas the Healthy class had lower values in these metrics. For the binary classification task, the Alzheimer and Healthy classes achieved an accuracy of 76.36% using the standard feature extraction method, whereas the modified feature extraction method achieved an accuracy of 99.71% as shown Table 13. Both classes showed lower recall, precision, and F1-scores with the standard method compared to the results obtained using the modified feature extraction method.

**Table 12:** Classification metrics for Alzheimer, frontotemporal, and healthy classes.

**Class**	**Precision**	**Recall**	**F1-score**	**Support**
Alzheimer	0.60	0.77	0.67	1,876
Frontotemporal	0.68	0.68	0.68	1,597
Healthy	0.60	0.33	0.43	1,106

**Table 13:** Classification metrics for Alzheimer and healthy classes.

**Class**	**Precision**	**Recall**	**F1-score**	**Support**
Alzheimer	0.76	0.81	0.79	1,876
Healthy	0.76	0.71	0.73	1,597

### 4.6 Comparison with existing ML and DL model

To gauge the performance of the proposed model, it has been compared with existing studies in Table 14. In Miltiadous et al. (18), the authors achieved an 83.28% accuracy with the DICE-net model, utilizing EEG denoising and extracting Band power and coherence features as key steps in feature engineering. In Kachare et al. (16), the STEADYNet model achieved 88.00% accuracy for AD vs. NC and 92.25% for FTD vs. NC. Using a dual-input strategy, the model employed convolutional and features are extracted from EEG data using max-pooling layers. The research explored binary and multi-class classification, reporting a 97.59% accuracy in the multi-class setting. The study (19) utilized a CNN with pre-trained weights, achieving an accuracy of 82.30%. EEG feature extraction was performed in both the time and frequency domains, while a Vision Transformer complemented the CNN by capturing global feature representations. The classification task distinguished between AD, FTD, and NC. Ma et al. (20), EEG data was used to classify AD and FTD, achieving an initial accuracy of 91.5%. After optimizing the feature set by eliminating unnecessary attributes, the accuracy increased to 96.6%. A support vector machine (SVM) model was utilized for binary classification between these groups (20).

**Table 14:** Model accuracy comparison with existing papers using dataset.

**Paper**	**Model**	**Accuracy**	**Feature engineering**	**XAI**
Ma et al. (20)	Support vector machine	91.5%	PHI	✗
Miltiadous et al. (18)	Dual-Input Convolution Encoder Network (DICE-net)	83.28%	Band power and coherence	✗
Kachare et al. (16)	STEADYNet	97.59%	✗	✗
Chen et al. (19)	Vision transformer + CNN	80.23%	frequency channels	✗
**This work**	**Proposed model**	**80.34%, 99.7%**	**Modified RBP**	✓

No prior research utilized explainable AI (XAI) or lightweight models. To address this, the proposed study introduces a hybrid deep learning model with efficient feature engineering and a reduced number of parameters, improving accuracy in binary and multi-class classification while integrating SHAP.

## 5 Explainable artificial intelligence

**Figure 6:**
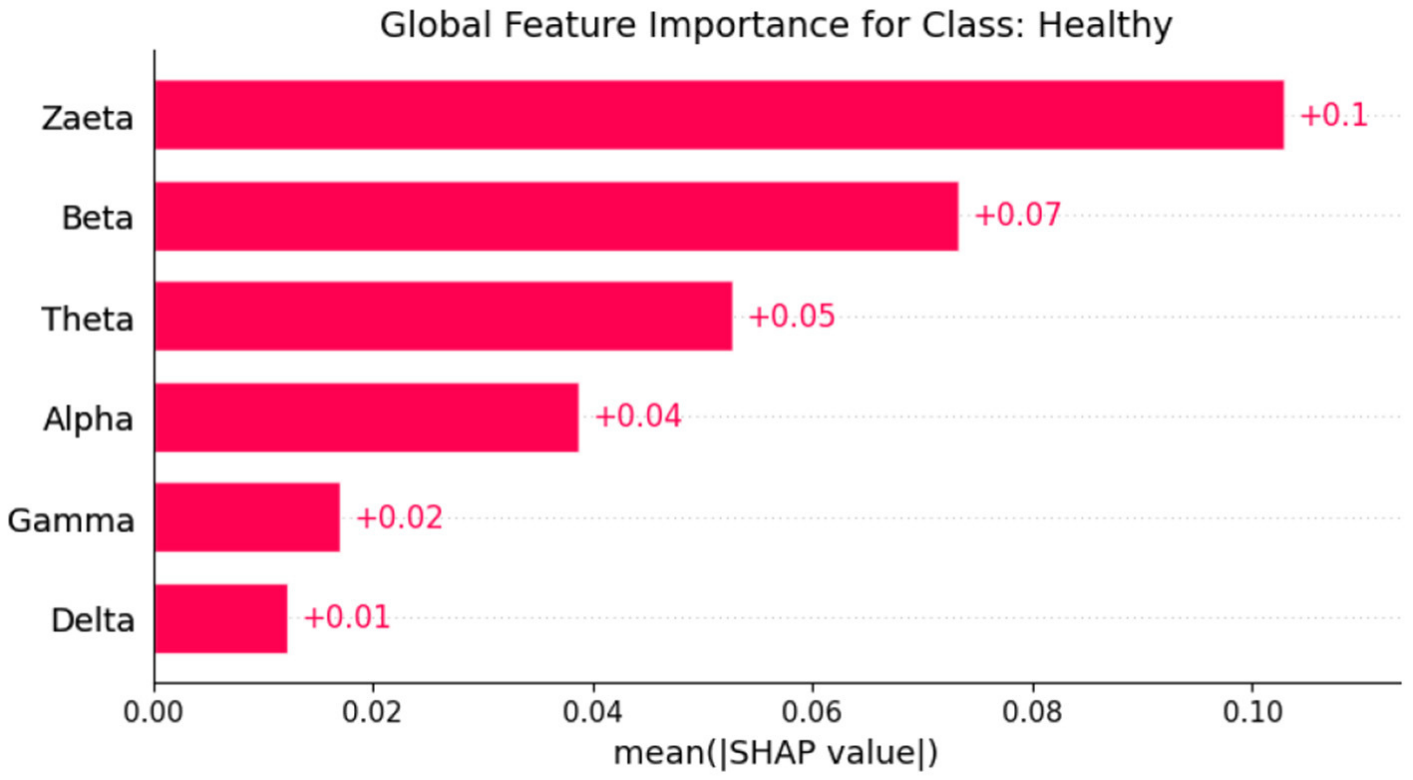
SHAP global feature importance graph for class healthy.

**Figure 7:**
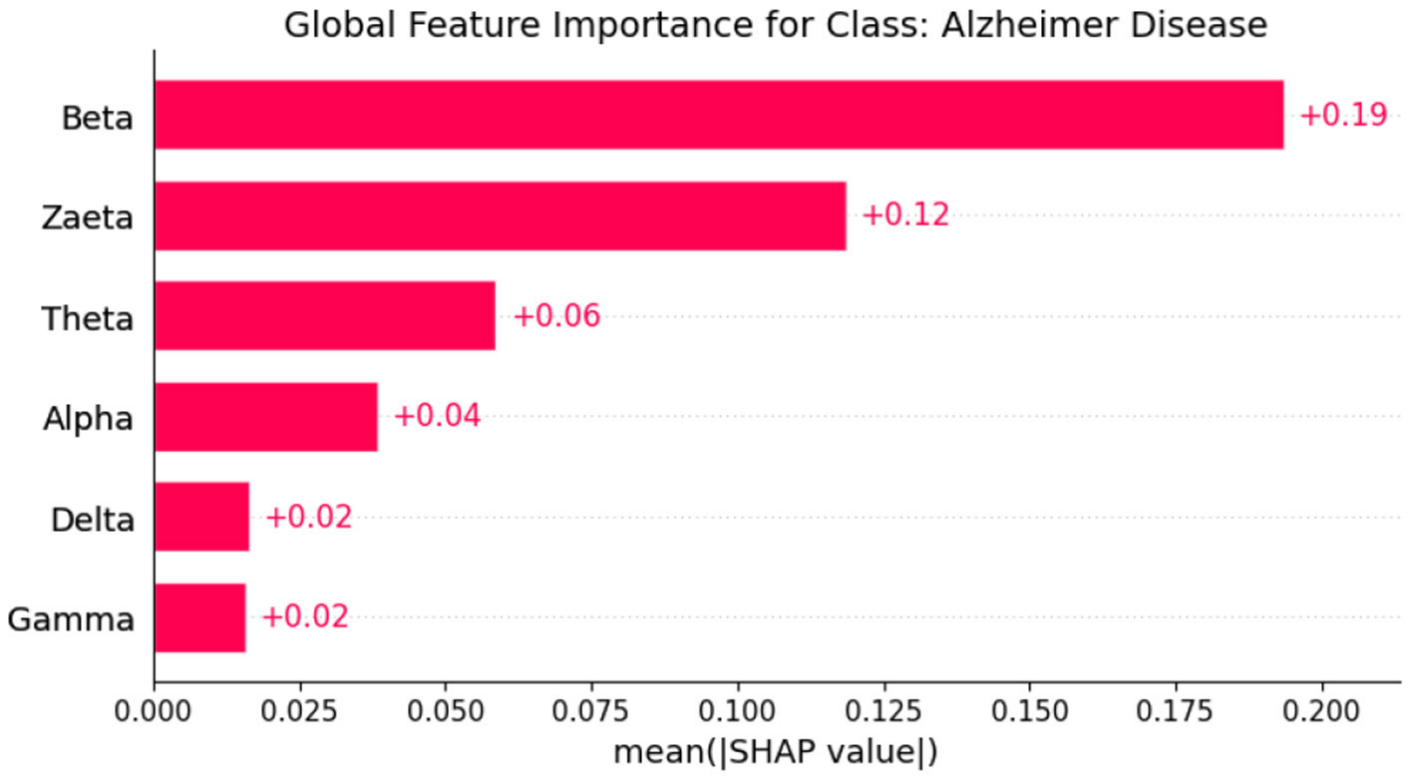
SHAP global feature importance graph for class Alzheimer's disease.

**Figure 8:**
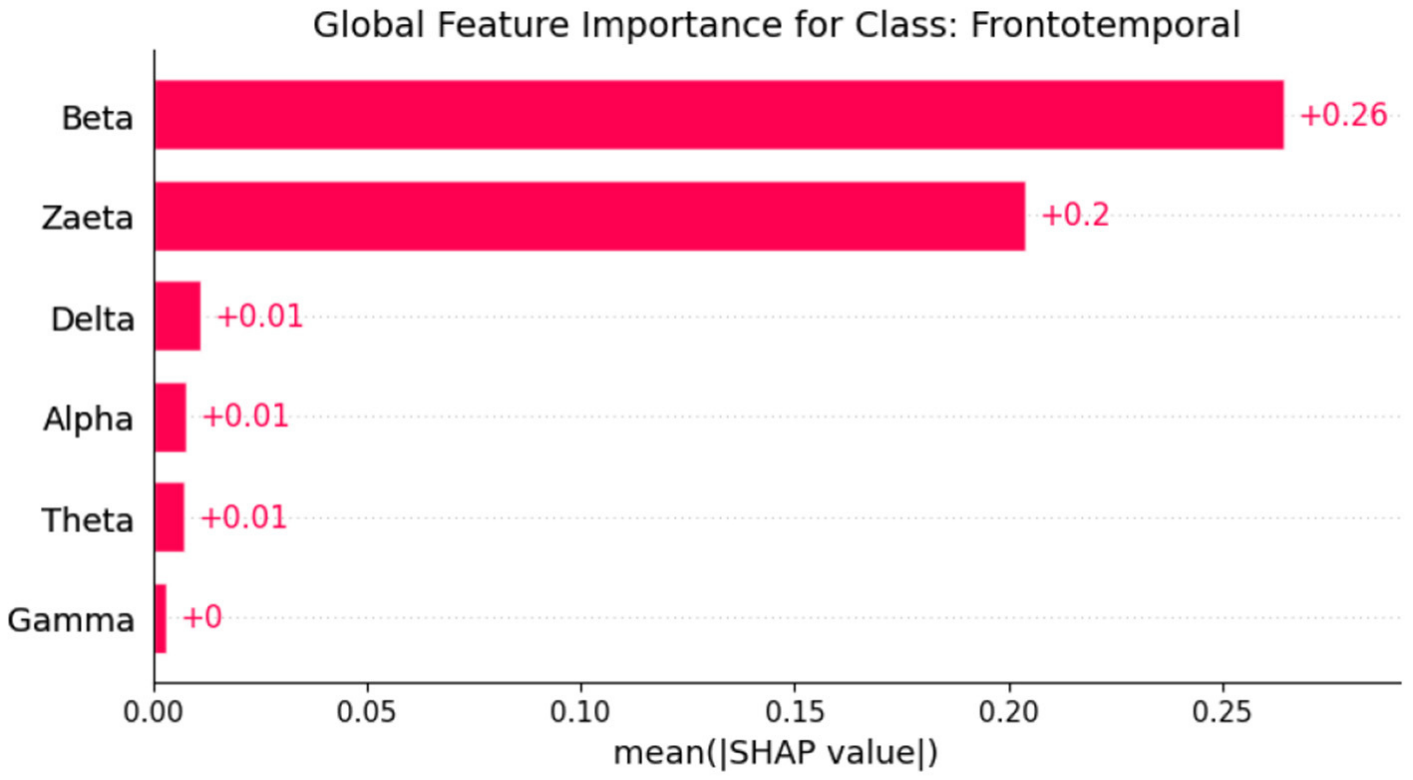
SHAP global feature importance graph for class frontotemporal disease.

Explainable Artificial Intelligence (XAI) is a crucial development in the field of artificial intelligence, focusing on making the decision-making processes of AI systems transparent and understandable to users. In medicine, the need for XAI is particularly significant due to the high stakes involved in clinical decision-making. Healthcare professionals require clear explanations for AI-driven recommendations to ensure trust and reliability in these technologies. By improving interpretability, XAI not only helps clinicians approach AI methods with caution but also fosters a deeper understanding of AI applications in medical practice, ultimately promoting data-driven and mathematically grounded medical education (21). The SHAP (SHapley Additive exPlanations) (22) global feature importance graphs depict the contribution of different frequency bands (Zaeta, Beta, Theta, Alpha, Delta, Gamma) to the classification of three classes: Healthy, Alzheimer's Disease, and frontotemporal Disease. In Figure 6, the SHAP values for the Healthy class show that Zaeta has the highest importance (+0.1), followed by Beta (+0.07) and Theta (+0.05). This indicates these frequency bands are most influential in predicting Healthy cases, while Alpha, Gamma, and Delta have minimal contributions. Figure 7 highlights the SHAP importance for Alzheimer's Disease, where Beta exhibits the highest importance (+0.19), followed by Zaeta (+0.12) and Theta (+0.06). These results suggest that Beta and Zaeta bands play a critical role in distinguishing Alzheimer's Disease from other classes. In Figure 8, the SHAP values for the Frontotemporal Disease class demonstrate that Beta has the most significant influence (+0.26), with Zaeta being the second most important feature (+0.2). The other frequency bands, including Theta, Alpha, Delta, and Gamma, contribute very minimally to this classification.

**Figure 9:**
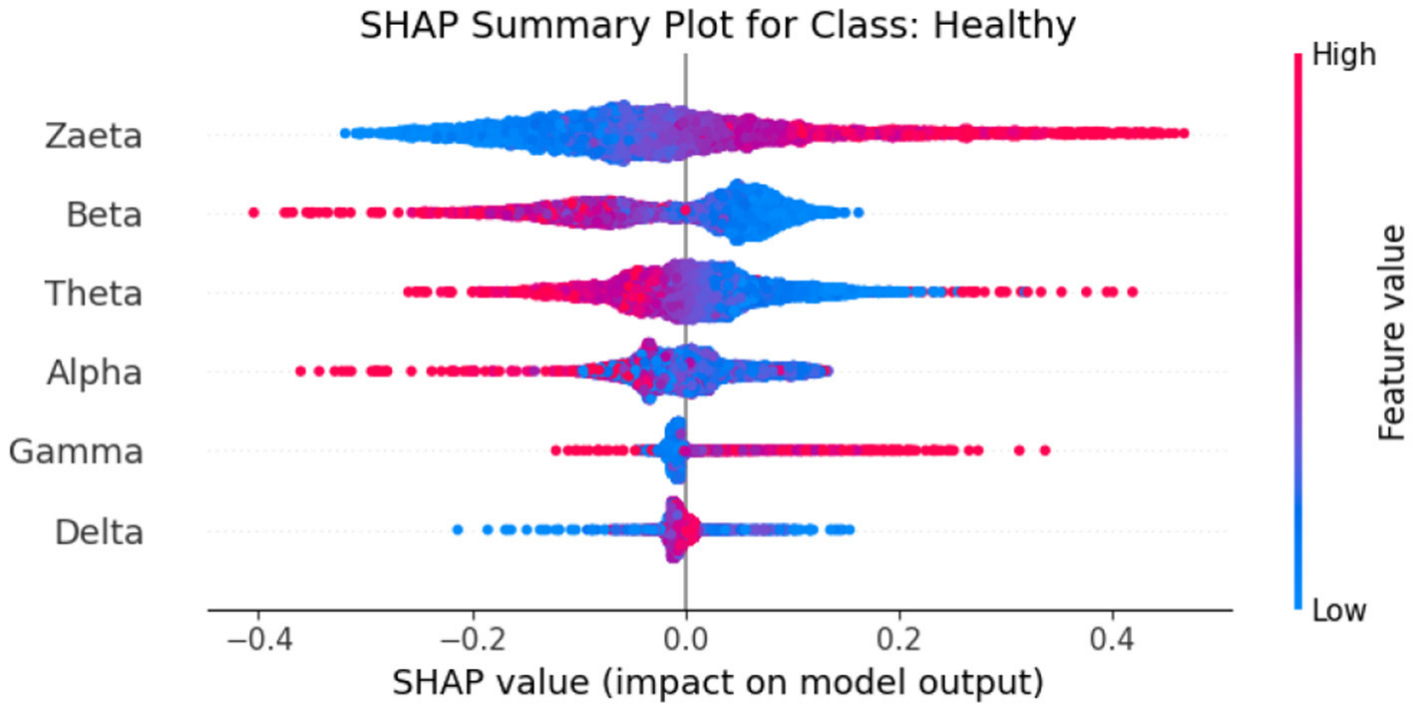
SHAP Summary graph for Class Healthy.

**Figure 10:**
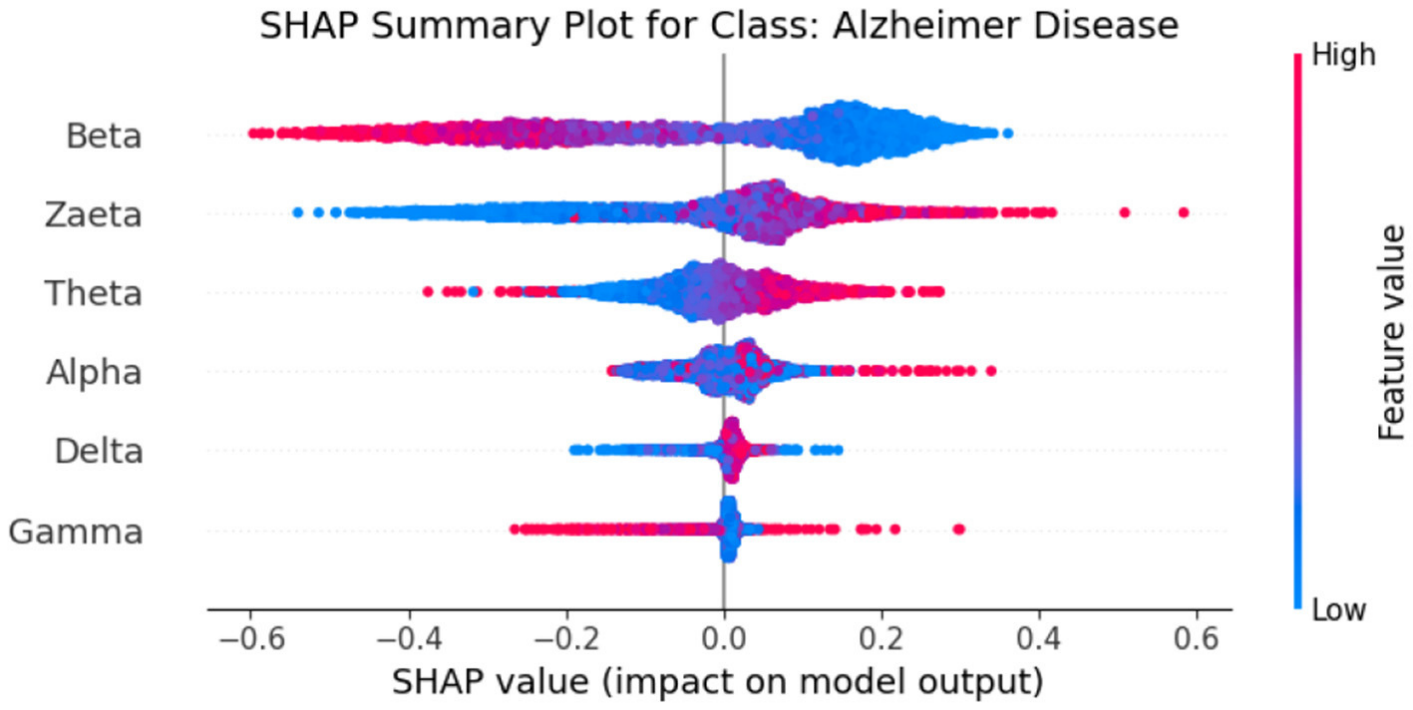
SHAP summary graph for class Alzheimer's Disease.

**Figure 11:**
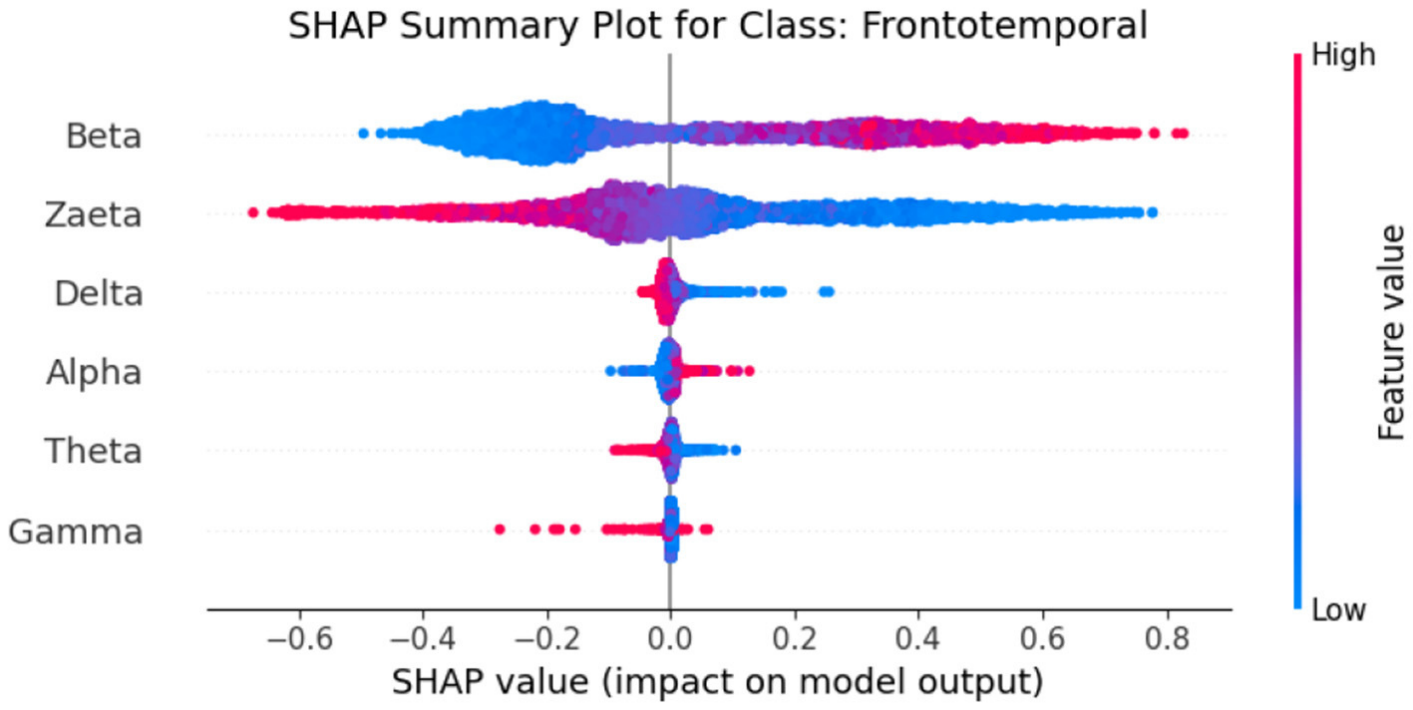
SHAP summary graph for class frontotemporal disease.

The SHAP summary graphs explain the contributions of different features to the predictions of a proposed hybrid deep learning model for three different Alzheimer's disease, and frontotemporal disease. Each plot shows the impact of the features on the model's output. The x-axis represents the SHAP values, indicating whether a feature contributes positively or negatively to the prediction for a specific class.The Healthy class plot Figure 9 shows distinct feature behavior compared to the disease classes. Here, the SHAP values indicate a different pattern of influence, with Zaeta and Beta waves also playing critical roles but in opposite directions from the disease classes. For the Alzheimer's Disease class Figure 10, features such as Beta and Zaeta wave characteristics show a stronger positive or negative influence on predictions, with higher feature values (red points) generally pushing predictions in one direction. In this plot Figure 11, the Zaeta and Beta waves seem to have the most significant influence on the model's predictions, with both high and low feature values affecting the SHAP values. The distribution of points along the x-axis for these features suggests that they are crucial in determining whether the prediction aligns with frontotemporal disease.

**Figure 12:**
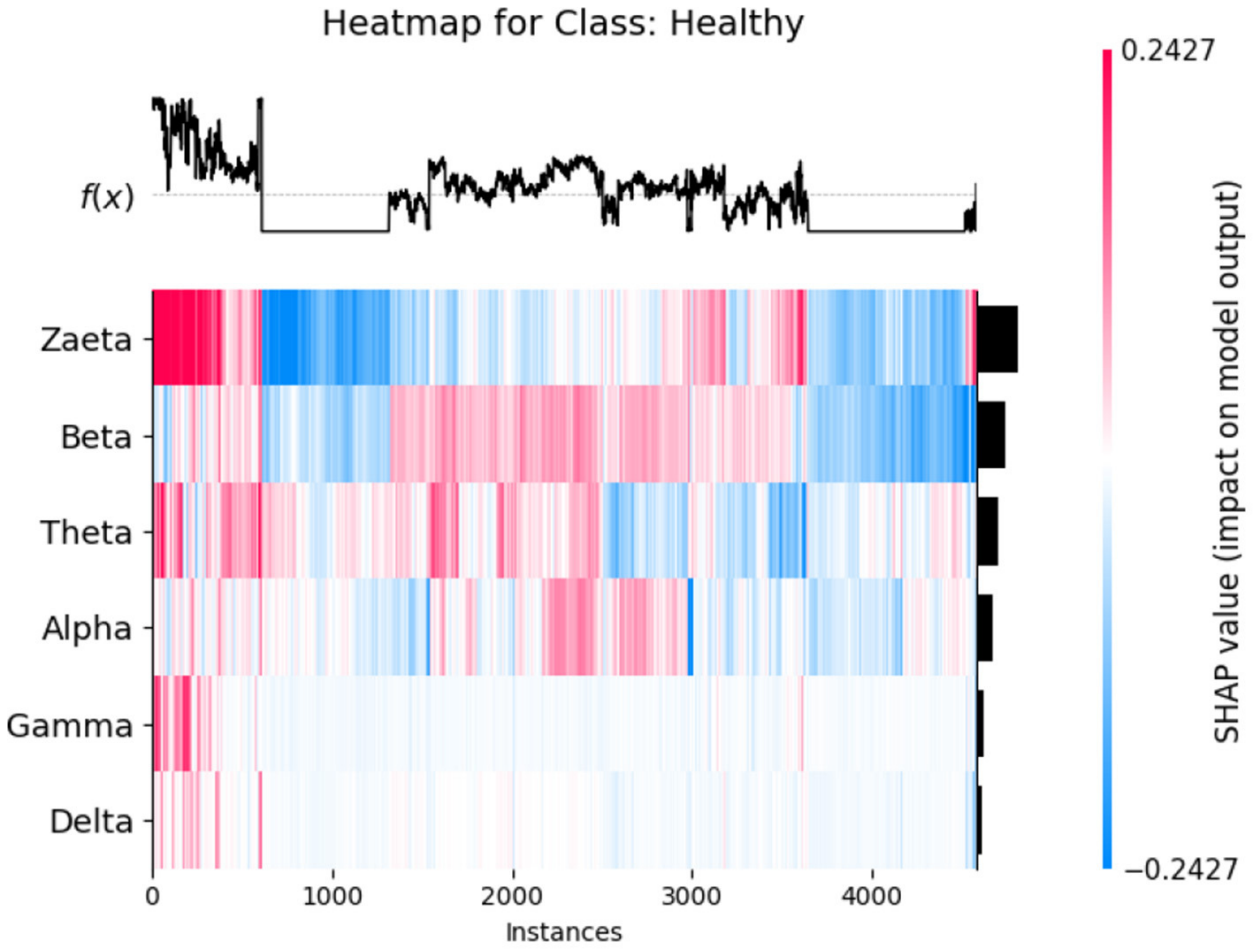
SHAP heatmap for class healthy.

**Figure 13:**
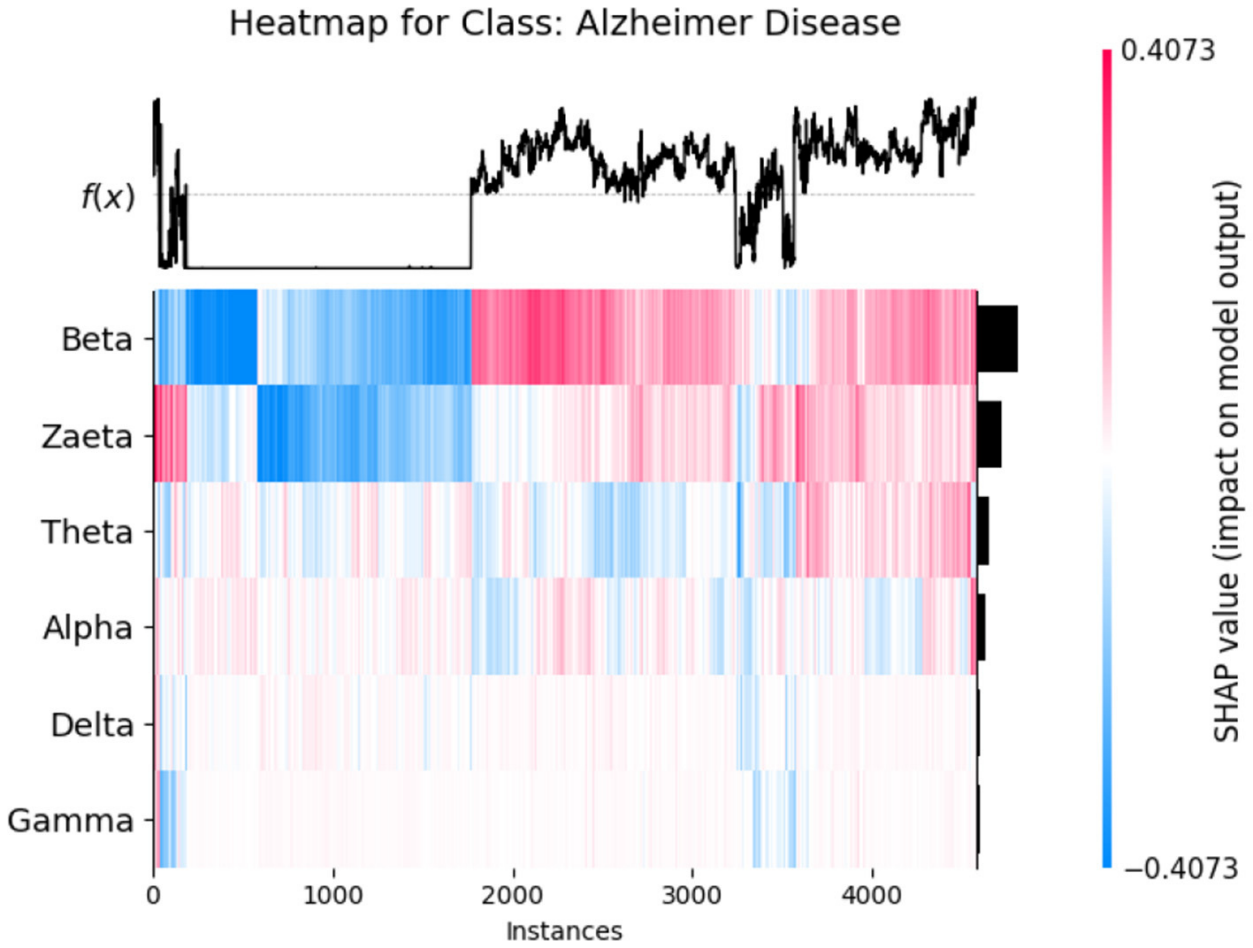
SHAP heatmap for class AD.

**Figure 14:**
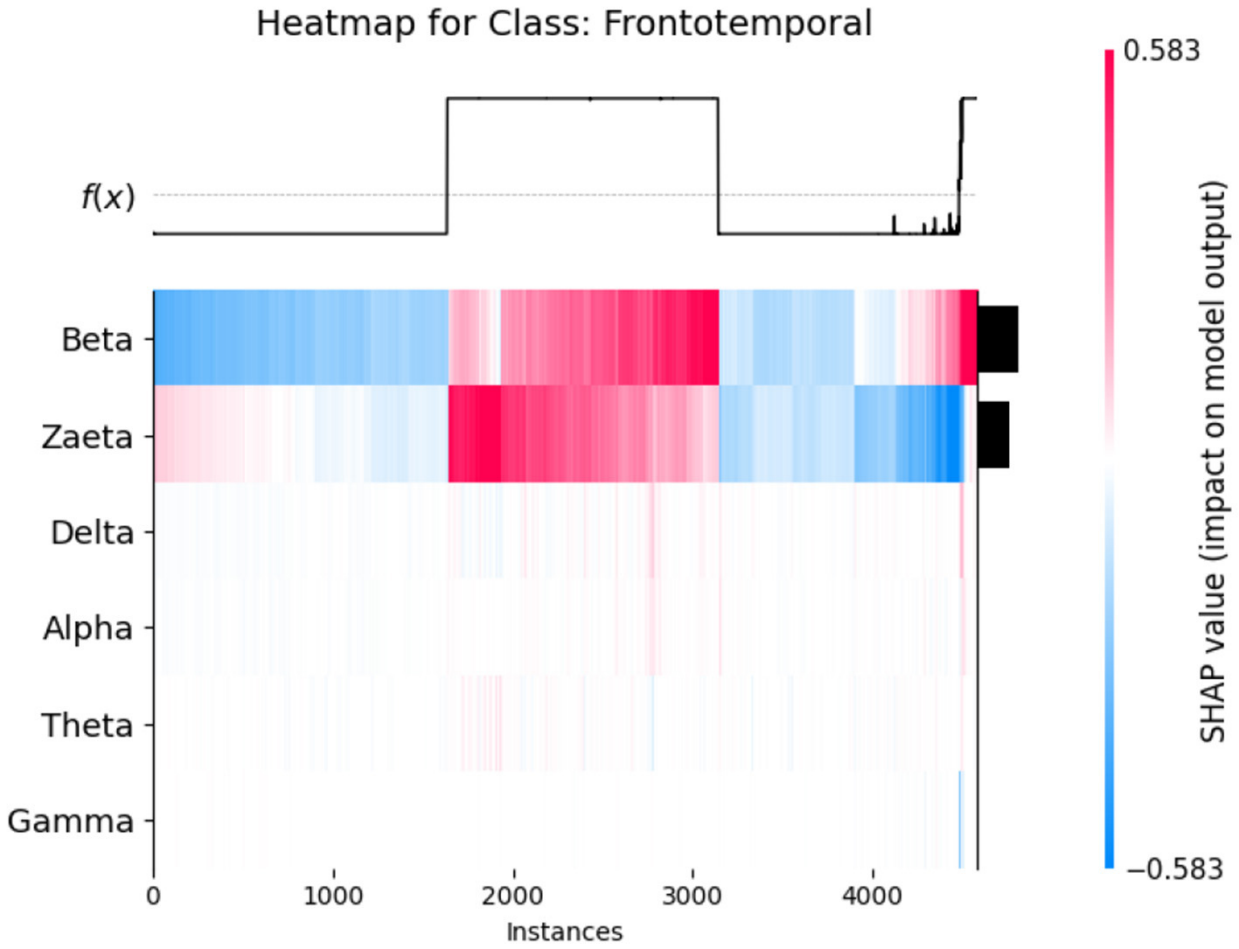
SHAP heatmap for class frontotemporal disease.

The SHAP heat maps show how different brain wave features contribute to the model's predictions for healthly, Alzheimer's Disease, and Frontotemporal Disease. Each row represents a feature, while the columns represent individual instances. Each model input's global importance is shown as a bar plot on the plot's right side. Beta and Zaeta waves are among the features that commonly display blue in Healthy class Figure 12, suggesting that they have a negative impact on the prediction and force the model to classify these phenomena as healthy. On the other hand, beta and Zaeta waves frequently show red in the AD class Figure 13, indicating that they are highly predictive of Alzheimer's disease. How these features adjust to different data points is seen in the mixed pattern across instances. For Frontotemporal Disease Figure 14, Beta and Zaeta waves again dominate with strong red contributions, emphasizing their importance for this class. Compared to Healthy, there are more concentrated positive contributions (red), pushing predictions toward the disease class. These heat maps reveal the nuanced role of brain wave features in distinguishing between healthy and diseased states.

### 5.1 Neurophysiological interpretation of frequency band importance

The SHAP visualizations in (Figures 6–14) reveal that the Zaeta and Beta frequency bands consistently exhibit high SHAP values across all classification tasks, indicating their dominant contribution in distinguishing between Alzheimer's Disease (AD), Frontotemporal Dementia (FTD), and healthy controls. This is not merely a data-driven outcome but has a neurophysiological basis grounded in clinical EEG studies.

The Beta band is associated with active cognitive processing, attention, and motor control. Abnormalities in Beta activity—particularly elevated or diminished power—have been reported in AD patients, often linked to disruptions in cognitive and executive functions. In contrast, FTD patients may exhibit distinct patterns in Beta activity due to altered frontal lobe functioning, which is characteristically impaired in FTD but less so in early AD.

The Zaeta band, though less commonly named in classical EEG literature, overlaps with the high Alpha to low Beta range and serves as a transitional band. Our modified Relative Band Power (RBP) analysis captures Zaeta as a distinct band, enabling finer differentiation. The elevated importance of Zaeta in our SHAP analysis suggests that subtle shifts in mid-frequency rhythms play a significant role in disease-specific EEG patterns. Specifically, such shifts may reflect compensatory mechanisms or region-specific slowing in cortical activity, both of which are documented phenomena in dementia-related neurodegeneration.

Therefore, the SHAP-derived feature dominance is consistent with known pathophysiological changes in brain activity across dementia subtypes. The model not only learns these discriminative patterns effectively but also explains them in a way that aligns with clinical neurophysiology, enhancing interpretability and potential clinical utility.

## 6 Conclusions and future direction

This paper addressed the critical need for an accurate and efficient detection of mental disorders, i.e., AD and (FTD). A lightweight TCN-LSTM hybrid model has been proposed for the aforementioned purpose. To prepare the data for experimentation, a modified Relative Band Power (RBP) analysis was performed to extract six EEG frequency bands via power spectrum density (PSD) computations. The proposed model achieved 99.70% accuracy for the classification of Frontotemporal Disease vs. Healthy, and 99.74% accuracy for Alzheimer vs. Healthy. In another binary task, where Alzheimer and Frontotemporal data were combined into a single class and classified against Healthy, the model achieved 99.80% accuracy. For the three-class classification, accuracy 80.34% achieved. Evaluation metrics including AUC-ROC, recall, confusion matrix, and F1-score were calculated for each classification. High scores were achieved across all multiclass categories, except the Healthy class, which showed reduced recall (35%) and F1-score (47%) as a result of data imbalance. Finally, the integration of SHAP for explainability further enhanced the model's transparency, making it a valuable tool for clinical applications. The proposed method proved to be an efficient and effective solution for the detection of AD and (FTD). Future research may include the use of large and diverse datasets focusing on the exploration of additional EEG characteristics Vascular, Lewy Body Dementia, and Creutzfeldt-Jakob Disease data can be used to train and validate the model with an XAI approach while maintaining patient data privacy and security.

## Data Availability

The original contributions presented in the study are included in the article/supplementary material, further inquiries can be directed to the corresponding author. The datasets analyzed and utilized for this study can be found at DOI: 10.3390/data8060095 and 10.18112/openneuro.ds004504.v1.0.5.
